# Assessment of the reliability of at-home caregiver-collected anthropometric measurements

**DOI:** 10.3389/fped.2024.1441321

**Published:** 2024-09-04

**Authors:** Jenny J. Ly, Ana Sosa, Matthew Heidman, Matthew F. Dixon, Christian Ostolaza, Susan M. Dallabrida

**Affiliations:** SPRIM PRO, Indian Harbour Beach, FL, United States

**Keywords:** anthropometry, infants, remote measurement, telemedicine, caregiver measurement, clinical trials

## Abstract

**Introduction:**

Anthropometric measurements provide valuable information about infant growth patterns and can help identify nutrition, growth, and developmental concerns. With the increasing use of telehealth and decentralized clinical trial approaches, there is potential for caregivers to collect anthropometric measurements at home via teleconference with healthcare providers (HCPs) to monitor infant growth, which indirectly reflects health status. This study aimed to evaluate whether telehealth-guided caregivers can utilize standardized methods and home-use measurement equipment to collect reliable anthropometric measurements compared to HCPs and study nurses.

**Methods:**

The study compared the weight, length, and head circumference measurements collected by caregivers (*n* = 8 pairs), pediatric HCPs (*n* = 7), and study nurses (*n* = 4), who served as the gold standard comparator group. Four silicone dolls with varied anthropometrics were used as surrogates for human infants.

**Results:**

Caregiver inter- and intra-observer technical errors of measurement (TEMs) were all equal to or below the maximum allowed error (MAE). For HCPs, only intra-observer TEM for length and inter-observer TEM for HC and length were within the MAE. There was no evidence of bias for either caregiver or HCP measurements compared to the gold standard. Coefficients of reliability (*R*) were greater than 0.96 for all measurements.

**Discussion:**

Preliminary results from this study demonstrate that telehealth-guided caregivers can capture accurate and reliable anthropometric measurements compared to HCPs. The results suggest that remote measurement collection allows for more frequent monitoring while reducing the burden on patients and caregivers in primary care and clinical trials such as infant formula growth monitoring studies.

## 1 Introduction

Anthropometric measurements of infants during their first year of life provide important information about their growth patterns and can help identify potential health concerns. Accurate serial measurements taken at routine well-child visits, including head circumference, length, and weight, are plotted on age- and gender-specific charts from the World Health Organization (WHO) or Centers for Disease Control and Prevention (CDC) (1). These measurements can reveal abnormal growth patterns that warrant further investigation and can aid in diagnosing malnutrition, stunting, wasting, congenital or acquired hormonal disturbances, neurological abnormalities, and other medical problems (2). Anthropometric measurements are also used to determine nutritional status and can identify infants under 6 months old who are at risk of mortality (3) due to malnutrition or neonatal adiposity (4, 5), which is linked to childhood obesity and metabolic syndrome (6, 7). Thus, anthropometric measurements can be utilized in primary care practice and pediatric clinical trials as a non-invasive, inexpensive, and efficient assessment tool for evaluating general health status of infants, to help identify potential nutrition, growth, and developmental issues (2, 8, 9). Frequent monitoring of anthropometric measurements can also help healthcare providers (HCPs) and researchers determine whether treatments or interventions are effective. In trials such as infant formula growth monitoring studies (GMSs), weight gain is the primary endpoint sought by the United States Food and Drug Administration (FDA), with total body length and head circumference as secondary endpoints (10, 11).

Traditionally, the collection of infant anthropometric measurements has been performed by trained HCPs and requires infants and their caregivers to visit pediatric clinics during well-child or sick visits. In clinical trials, caregivers are required to visit the study site with their infant (the participant) during specific time windows dictated by the study protocol. For example, GMSs require a site visit three times within a 4-week period and every 2 weeks thereafter (11), posing a significant burden on both patients and caregivers. The decentralized clinical trial (DCT) approach, leveraging telehealth technology, offers the potential to alleviate some of this burden. Over the last decade, as telecommunication technology and connectivity have advanced, remote visits for primary care, specialty care, and clinical trials have become more ubiquitous (12). For example, telehealth has been used to observe children's developmental skills and track neurodevelopment (13), conduct remote physical exams (14), and monitor chronic conditions (15). Remote telehealth visits can reduce the burden on patients and caregivers by reducing costs, time, and transportation needs, expanding access to specialists through remote consultations, and removing language barriers in under-resourced communities (16). In addition, in clinical trials, a DCT approach can improve accessibility and increase the inclusion of a more diverse study population (17, 18).

The FDA has recently approved the execution of GMS protocols utilizing DCT methodologies to collect anthropometric data in real time outside of study sites. Specifically, caregivers were provided with infant weight scales, length mats, and head circumference tapes to collect measurements at home while teleconferencing with study staff who guided them through the measurement process.

Caregiver-reported measurements have been validated in children over the age of 6 months (19) and remotely by caregivers in children over 2 years old (20). However, to date, the reliability of anthropometric measurements collected by caregivers in infants younger than 6 months has yet to be investigated. Inaccurate or missing measurements may lead to missed crucial interventions, unnecessary referrals, or heightened parental concerns. The objective of this study was to evaluate whether caregivers, guided by study nurses via audiovisual teleconference calls, can utilize standardized methods and home-use measurement equipment to collect reliable anthropometric measurements compared to HCPs in a clinical setting and research study nurses. Results from the study will help pediatric practitioners and researchers determine whether at-home caregiver collection of anthropometric measurements is a feasible alternative to in-clinic measurements by HCPs.

## 2 Methods

### 2.1 Study design

This study compared the reliability of two methods for collecting anthropometric measurements in infants: telehealth-guided caregivers using home measurement equipment versus trained HCPs in a clinical setting. Anthropometric measurements collected by study nurses served as the reference or “gold standard” measurements. Four silicone, non-vinyl dolls of varied sizes and anthropometric characteristics representing newborn infants were measured in place of human infants. The use of infant dolls ensured that all participants measured a standardized study subject/object with identical anthropometric values to evaluate reliability across the three groups. Human infant measurements, especially weight, can fluctuate over hours due to feeding and/or voiding waste. In addition, infant behaviors (e.g., general movement, crying, or wriggling) can have a high impact on consistency between measurements; thus, standardized infant dolls were utilized. The study was reviewed by Sterling Institutional Review Board (IRB) and determined to be exempt from the requirements of IRB approval and informed consent, as it met the U.S. Department of Health and Human Service Category 2 Exemption criteria.

### 2.2 Study participants

Anthropometric measurements were collected by three groups of participants: (1) caregivers, (2) HCPs, and (3) study staff nurses. The caregiver group (*n* = 8 pairs) consisted of individuals without any healthcare experience or experience collecting anthropometric measurements. The 16 caregivers worked in pairs following the guidelines of the American Academy of Pediatrics for measuring recumbent length in infants (21). The HCP group (*n* = 7) consisted of board-certified, registered pediatric nurses at the Garden City Pediatrics in Beverly, MA, USA. The third group, study nurses at SPRIM PRO (*n* = 4), comprised board-certified, registered pediatric nurses.

### 2.3 Materials

Four different sizes of silicone, non-vinyl infant dolls were utilized for this study. Anthropometric measurements were captured and recorded, including head circumference (cm, to the nearest 0.1 cm), length (cm, to the nearest 0.5 cm), and weight (kg, to the nearest 0.005 kg). Each anthropometric measurement was captured in duplicate.

### 2.4 Procedures

Each pair of caregivers was shipped the four silicone infant dolls and home-use measurement equipment (i.e., head circumference measurement tape, infant length measurement mat, and tabletop digital infant weight scale) to collect anthropometric data from the dolls. Caregivers were given instructions and guidance during measurement collection via a video conference call with a study nurse who did not take part in providing the gold standard anthropometric measurements. The study nurse watched and guided caregivers in doll manipulation, device operation, and measurement reading. For head circumference measurements, the study nurse provided instructions on head circumference tape preparation, proper tape placement on the head and subsequent adjustments, and accurate reading of the tape measurement in centimeters. Guidance for infant length measurement included mat preparation, proper head placement and manipulation of the legs and feet of the dolls, movement of the footboard, and accurate reading of the mat measurement in centimeters. Guidance for weight measurements included taring of the scale, proper placement of the doll on the scale, utilization of the stabilizing feature, and accurate reading of the digital output in kilograms. Caregivers worked in pairs to collect the anthropometric measurements and read the measurements out loud for the study nurse to record the data.

Pediatric HCPs were shipped and measured the same four silicone infant dolls. However, they used their in-clinic measurement equipment and were not provided with the standardized training and guidance the caregivers were given. Instead, they captured anthropometric measurements based on their clinical training and the best practices of their clinics. The HCPs captured and recorded measurements in duplicate on a paper form to submit to the study staff.

As the gold standard reference group, study nurses were shipped the same four silicone dolls and measurement equipment as the caregivers. They captured the infant doll anthropometric measurements at home following the same standardized instructions as the caregivers but were not observed via conference calls. Their measurements were recorded in duplicate on a paper form and submitted to the study staff.

### 2.5 Statistical analysis

All anthropometric measurements are subject to human error, and repeated measurements can result in technical variability. Measurement reliability is a direct indicator of data quality. In this study, intra- and inter-observer technical error of measurement (TEM), average bias relative to the gold standard, and coefficient of reliability (*R*) were calculated for the three anthropometric measurements in accordance with reliability analysis standards used in anthropometric studies, including the Multicenter Growth Reference Study (MGRS) of the WHO (22). The results were interpreted based on these standards. In addition to *R*, the intraclass correlation coefficient (ICC) for the three measurements was also calculated.

*TEM* is an accuracy index that measures the variability of the same measurement and is a common way to express the error margin in anthropometry. This study examined both intra-observer reliability, which refers to the variability of repeated measurements performed by the same observer, and inter-observer reliability, which refers to the variability of measurements performed by different observers in the same group. For its interpretation, TEM values were considered “acceptable” when they fell within ±2 times the gold standard TEM (22).

Intra-observer TEM measurements were calculated with the following formula generalized for K observers:

$$\mathrm{Intra}\text{-}\mathrm{observer}\;\mathrm{TEM}=\sqrt{\frac{\sum_{\;j=1}^{K}\left(\sum_{i=1}^{N}(M_{ij1}-M_{ij2})\right)}{2N}}$$

where $M_{i1}$ and $M_{i2}$ are the two repeated measures taken by each observer *j* for the ith study object (silicone infant doll), *N* represents the number of study objects, and *K* is the number of observers taking measurements by groups: caregivers (*n* = 8), HCPs (*n* = 7), and gold standard nurses (*n* = 4).

The inter-observer TEM was calculated as follows (22):

$$\mathrm{Inter}\text{-}\mathrm{observer}\;\mathrm{TEM}=\sqrt{\frac{1}{N}\overset{N}{\underset{i=1}{\sum }}\frac{1}{K_{i}-1}\left|\overset{K_{i}}{\underset{\;j=1}{\sum }}Y_{ij}^{2}-\frac{{\left(\sum_{\;j=1}^{K_{i}}Y_{ij}\right)}^{2}}{K_{i}}\right|}$$

where $Y_{ij}$ is one of the duplicated measurements taken by observer *j* for study object *i* (just the first recorded measurement was selected), $K_{i}$ represents the number of observers that measured study object *i*, and *N* is the number of study objects measured.

*Average bias* is the average difference between measurements taken by the gold standard (study nurse) group and those by the HCPs and caregivers. It is commonly used to determine whether the HCPs and/or caregivers systematically over- or under-estimated their measurements depending on a positive-signed or a negative-signed bias, respectively. It was calculated by the following formula (22):

$$\mathrm{Average}\;\mathrm{bias}=\frac{\sum_{i=1}^{N_{G}}\left[\left(\sum_{\;j=1}^{K}\frac{\left(M_{ij1}+M_{ij2}\right)}{2K}\right)-\left(\sum_{g=1}^{L}\frac{\left(M_{ig1}+M_{ig2}\right)}{2L}\right)\right]}{N_{G}}$$

where $M_{ij1}$ and $M_{ij2}$ are the duplicated measurements recorded by observers *j* in caregiver and HCP groups for the ith study object, and $M_{ig1}$ and $M_{ig2}$ are the duplicated readings taken by observers,g of the gold standard group for the study object *i*, $N_{G}$ is the number of study objects measured by the expert, *K* is the number of observers measuring the same study object (K=8 for caregivers and K=7 for HCPs), and *L* represents the number of experts measuring the same study object in the gold standard group (L=4). Average bias was considered “acceptable” if it was between ± 2.8 times the gold standard TEM (22).

The *coefficient of reliability* (*R*) estimates the proportion of variance due to true differences rather than measurement errors. *R* ranges from 0 to 1, with ≥0.8 indicating excellent reliability and 0.61–0.8 indicating substantial reliability. The coefficient of reliability was calculated as follows:

$$R=1-\frac{{\left(\mathrm{inter}\text{-}\mathrm{observer}\;\mathrm{TEM}\right)}^{2}}{SD^{2}}$$

where inter-observerTEM was calculated as explained before and standarddeviation(SD) was calculated for each anthropometric variable for the silicone infant dolls.

*Intraclass correlation* (*ICC*) assesses reliability by comparing the variability of different measurements made by the same observer to the total variation across all measurements and all observers.

The ICC was calculated by the following formula:

$$ICC=\frac{\sigma^{2}(b)}{\sigma^{2}(b)+\;\sigma^{2}(w)}\;$$

where *σ*^2^(*w*) is the pooled variance within observers and *σ*^2^(*b*) is the variance between observers. ICC also ranges from 0 to 1, with values >0.9 indicating excellent reliability, between 0.75 and 0.9 indicating good reliability, between 0.5 and 0.75 indicating moderate reliability, and <0.5 indicating poor reliability.

## 3 Results

### 3.1 Demographics

A total of 27 individuals participated in the study: 16 caregiver participants in pairs (*n* = 8) with no experience in healthcare or anthropometrics; 7 pediatric HCPs with an average of 5 years of experience (SD, 3.9) at the Garden City Pediatrics in Beverly, MA; and 4 study nurses with an average of 10 years of experience (SD, 6.34) in taking infant anthropometrics.

### 3.2 Descriptive data

The average and standard deviation of measurements for head circumference (cm), length (cm), and weight (kg) for each of the four study objects (infant dolls) by each group of observers are listed in Table 1. Intra- and inter-observer variabilities are described in detail in the next section.

**Table 1:** Head circumference, length, and weigh of study objects by the observer group.

		Head circumference (cm)			Length (cm)			Weight (kg)		
Study object		Gold standard (*n* = 4)	HCP (*n* = 7)	Caregiver (*n* = 8)	Gold standard (*n* = 4)	HCP (*n* = 7)	Caregiver (*n* = 8)	Gold standard (*n* = 4)	HCP (*n* = 7)	Caregiver (*n* = 8)
SO-1	Mean	32.47	31.69	32.44	46.75	47.82	46.91	2.766	2.758	2.755
	SD	0.23	0.51	0.46	0.87	0.52	0.81	0.002	0.006	0.009
SO-2	Mean	28.37	27.59	28.42	38.00	39.43	38.52	1.682	1.698	1.692
	SD	0.24	0.41	0.42	0.00	0.81	0.52	0.002	0.009	0.003
SO-3	Mean	33.90	33.20	33.91	47.62	49.50	47.31	3.754	3.742	3.750
	SD	0.20	0.45	0.36	0.52	1.77	1.13	0.004	0.005	0.003
SO-4	Mean	37.85	37.53	38.12	56.71	58.71	57.14	5.372	5.331	5.363
	SD	0.38	0.36	0.69	1.06	1.36	0.90	0.058	0.010	0.018

SD, standard deviation; SO, study object.

Means by the observer group of head circumference (cm), length (cm), and weight (kg) of each study object (infant model) were calculated using the average of the two repeated measurements taken by each observer.

### 3.3 Technical error of measurement

**Figure 1:**
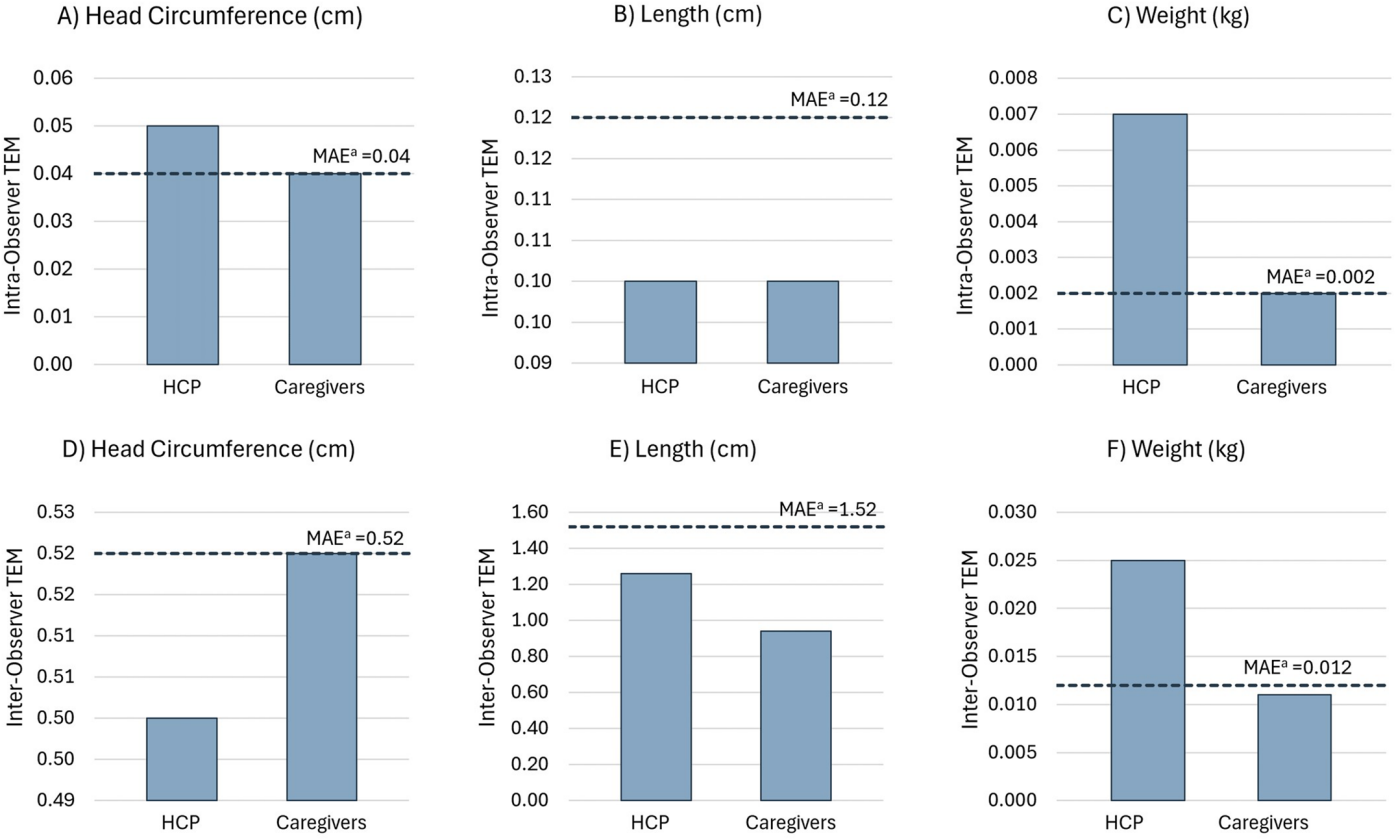
Intra-observer TEM for **(A)** head circumference, **(B)** length, and **(C)** weight. Inter-observer TEM for **(D)** head circumference, **(E)** length, and **(F)** weight. ^a^MAEs for intra- and inter-observer TEMs for the HCP and caregiver groups were calculated as two times the gold standard TEM (22).

Overall, for the three groups, intra-observer TEM values ranged 0.02–0.05 cm for head circumference, 0.06–0.10 cm for length, and 0.001–0.002 kg for weight (Table 2). All caregiver TEM values were equal to or below the maximum allowed error (MAE, Figures 1A–C) or two times the gold standard TEM, which represents 95% precision (22). However, for HCP, only TEM values for head circumference and weight were above the MAE (Figures 1A,C).

**Table 2:** Intra-observer and inter-observer technical error of measurement and coefficient of reliability (*R*) by the observer group.

	Intra-observer TEM			Inter-observer TEM			*R*		
	Gold standard (*n* = 4)	HCP (*n* = 7)	Caregiver (*n* = 8)	Gold standard (*n* = 4)	HCP (*n* = 7)	Caregiver (*n* = 8)	Gold standard (*n* = 4)	HCP (*n* = 7)	Caregiver (*n* = 8)
Head circumference (cm)	0.02	0.05	0.04	0.26	0.50	0.52	0.99	0.97	0.97
Length (cm)	0.06	0.10	0.10	0.76	1.26	0.94	0.98	0.96	0.98
Weight (kg)	0.001	0.007	0.02	0.006	0.025	0.011	1.00	0.99	0.99

TEM, technical error of measurement; *R*, coefficient of reliability.

For the three groups, inter-observer TEM values ranged 0.26–0.52 cm for head circumference, 0.76–1.26 cm for height, and 0.006–0.011 kg for weight (Table 2). All inter-observer TEM values were below the MAE, except for weight estimation by HCPs (Figures 1D–F). Moreover, the inter-observer TEM was greater than the intra-observer TEM for all measurements, indicating that the variability between observers was higher than that between the repeated measurements taken by each observer.

For caregivers, inter- and intra-observer TEM estimates for all measurements were within the MAE limit or 95% precision margin (Figure 1) and can be considered “acceptable” based on anthropometric study standards. However, for HCPs, only the intra-observer TEM for length (0.10 cm) and the inter-observer TEM for head circumference (0.50 cm) and length (1.26 cm) were within the MAE or “acceptable” range.

### 3.4 Average bias

**Figure 2:**
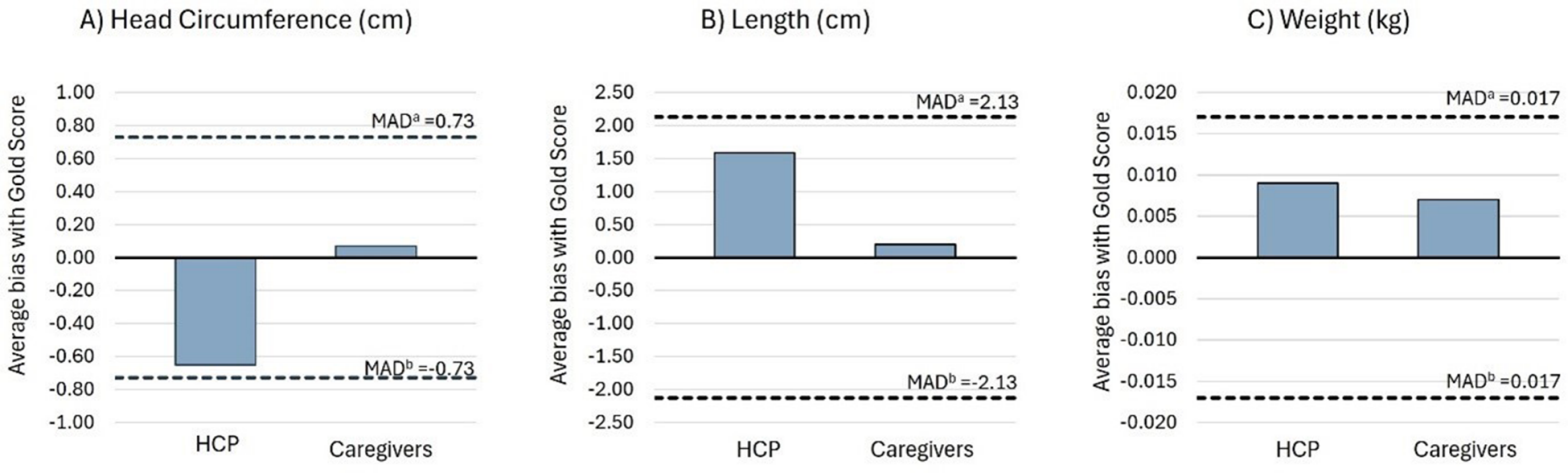
Average bias of the HCP and caregiver groups compared to the gold standard for **(A)** head circumference, **(B)** length, and **(C)** weight. ^a^Positive limit of the MAD between the gold standard with the HCP and caregiver groups. MAD limits were calculated as 2.8 times gold standard inter-observer TEM (22). ^b^Negative limit of the MAD between the gold standard with the HCP and caregiver groups.

Average bias estimates for all measurements were within the limits of the maximum allowed difference (MAD) or 2.8 times the gold standard inter-observer TEM and considered “acceptable” (22). According to the signs of average bias, HCPs tend to underestimate, while caregivers overestimate head circumference compared to the gold standard. For length and weight, both HCPs and caregivers tend to overestimate measurements compared to the gold standard (Figure 2).

### 3.5 Reliability of measurements data

Coefficients of reliability (*R*) were calculated for each variable and group of observers. All of them were above 0.96, indicating that 96% of the total variability was attributable to natural variation, and the remaining 4% represented the variability due to measurement error (Table 2). ICCs were also calculated for each variable and group of observers. Similarly, all values were above 0.98, indicating excellent reliability.

## 4 Discussion

Preliminary results from this study demonstrated that caregivers, under the guidance of study nurses via telehealth, can capture accurate and reliable anthropometric measurements at home. Standard anthropometric reliability analysis showed that intra- and inter-observer TEM values for all three measurements by caregivers were within the MAE limits (i.e., equal to or below twice the gold standard TEM values or 95% precision margins) and can be interpreted as “acceptable” based on anthropometric study standards (22). For HCPs, only the intra-observer TEM for length and the inter-observer TEM for head circumference and length were within the MAE or “acceptable” limits. Average bias estimates, *R* values, and ICC values for all three measurements, for both the caregiver and HCP groups, were within “acceptable” limits (Figure 2). This suggests that caregivers, utilizing the same measurement equipment and standardized training, under the supervision of a study nurse via telehealth, were as precise in their measurements compared to HCPs in a clinical setting.

Findings from this study add to the literature demonstrating good reliability and acceptable intra- and inter-observer TEM in anthropometric measurements collected by trained pediatric primary care providers (23), research staff (24, 25), and caregivers (26, 27). Even though studies showed that caregiver-collected measurements were overall accurate and reliable, some studies showed that caregivers were likely to underreport the height and weight of their children (26, 27). However, caregivers in those studies were not provided with training or supervision during measurement collection. Other studies have found that, with training and instructions, caregivers can collect anthropometric measurements of children as young as 6 months old with equivalent reliability to that of research staff (19, 28) and were able to classify the nutritional status of their children with good sensitivity and specificity (29). Similarly, this study found that with sufficient training as well as observation and guidance during video conference calls, caregivers were able to collect measurements with excellent reliability and “acceptable” intra- and inter-observer TEMs. Proper guidance and training are crucial in ensuring measurement accuracy and reliability, which is why the WHO recommends standardized measurement techniques, equipment calibration, and training on the proper operation of measurement devices, measurement reading, and manipulation of the infant (22).

Routine measurement collection during well-child visits is recommended to screen for malnutrition (3), obesity, or being overweight (23). Accurate and reliable caregiver-collected anthropometric measurements can allow for more frequent measurement collection. More frequent measurements can not only help identify neonatal adiposity and early-life risk factors that may lead to metabolic syndrome and insulin resistance later in life (30) but may also increase the likelihood of timely treatments and facilitate monitoring the progress of interventions to potentially prevent diseases later in life (7). The ability for caregivers to capture accurate measurements coupled with the increasingly widespread use of telehealth poses the potential to reduce patient and caregiver burden, provide less expensive care (16), and improve accessibility to clinical trials.

### 4.1 Limitations

The study results should be considered preliminary, with several limitations noted, including the small sample size. While the results suggest that anthropometric measurements taken by telehealth-guided caregivers are accurate and reliable, additional studies with larger sample sizes are needed to confirm the findings. The second limitation is the use of infant dolls as surrogates for human infants to ensure consistent anthropometric values for comparison across the three participant groups. The researchers acknowledge that collecting measurements from dolls differs from measuring live infants, which could add more variability due to feeding and/or voiding waste and infant movements, leading to higher TEM values. Future studies should also include measurement collection with human infants to confirm that the caregiver's intra- and inter-observer TEM values remain within acceptable limits despite the added variability. Third, the equipment, including the measurement tapes, mats, and scales used in the study, were the same for the gold standard (study nurse) and caregiver groups, while the HCP group used the equipment from their daily practice. The use of different in-clinic equipment by HCPs could have contributed to increased variability, although the practice reflects real primary practice. Future studies should assess whether, and to what extent, differences in equipment contribute to variability. Finally, the researchers acknowledge that while measurement equipment are typically provided in clinical trials at no cost to caregivers, it may not be economically feasible to provide equipment to all infants or require caregivers to purchase the equipment. Therefore, at-home caregiver measurement collection may be more suitable in cases where early-life risk factors have been identified and require frequent monitoring.

## 5 Conclusion

Overall, the preliminary results from this study indicate that telehealth-guided caregivers can use standardized methods and home-use measurement equipment to collect accurate and reliable anthropometric measurements, comparable to those collected by HCPs in a clinical setting. These findings support the continued use of this methodology in clinical trials, such as infant growth monitoring studies. This approach allows for more frequent monitoring while reducing the burden on patients and caregivers, providing more robust and accurate data sets.

## Data Availability

The raw data supporting the conclusions of this article will be made available by the authors without undue reservation.
